# Tumour microvessel density as predictor of chemotherapy response in breast cancer patients

**DOI:** 10.1038/sj.bjc.6600325

**Published:** 2002-06-17

**Authors:** O Tynninen, J Sjöström, K von Boguslawski, N O Bengtsson, R Heikkilä, P Malmström, B Østenstad, E Wist, V Valvere, E Saksela, T Paavonen, C Blomqvist

**Affiliations:** Haartman Institute, Department of Pathology, University of Helsinki, 00014 Helsinki, Finland; Department of Oncology, Helsinki University Central Hospital, 00029 Helsinki, Finland; HUCH Laboratory Diagnostics, Division of Pathology, Helsinki University Central Hospital, 00029 Helsinki, Finland; University Hospital, S-901 85 Umeå, Sweden; Rogaland Central Hospital, N-4011 Stavanger, Norway; University Hospital, S-211 85, Lund, Sweden; Ullevål Hospital, N-0407 Oslo, Norway; University Hospital of Tromsoe, Norway; Estonian Cancer Centre, Tallinn EE 00106, Estonia; University Hospital of Uppsala, S-75185 Uppsala, Sweden

**Keywords:** microvessel density, chemotherapy, metastatic breast cancer, predictive factor

## Abstract

The aim of this study was to evaluate the predictive value of intratumoural microvessel density in breast cancer. We studied immunohistochemically primary tumours of 104 patients with metastasised breast cancer who took part in a randomised multicentre trial comparing docetaxel to sequential methotrexate and 5-fluorouracil. Vessels were highlighted with factor VIII staining and counted microscopically. Microvessel density was compared with clinical response to chemotherapy and patient survival. The microvessel density of the primary tumour was not significantly associated with patient's response to chemotherapy, time to progression or overall survival in the whole patient population or in the docetaxel or methotrexate and 5-fluorouracil groups. However, disease-free survival was longer in patients with low microvessel density (*P*=0.01). These findings suggest that microvessel density of the primary tumour cannot be used as a predictive marker for chemotherapy response in advanced breast cancer.

*British Journal of Cancer* (2002) **86**, 1905–1908. doi:10.1038/sj.bjc.6600325 www.bjcancer.com

© 2002 Cancer Research UK

## 

Currently most breast cancer patients suffering from metastatic disease receive chemotherapy and/or hormone therapy. Curative treatment is not yet available for metastatic breast cancer and its management is palliative in almost all cases. Chemotherapy is frequently toxic and only half of the patients with metastatic disease usually respond to the treatment. There is a need for predictive markers for chemoresistant tumours in order to avoid unnecessary side effects of chemotherapy.

Hormonal receptor status and tumour cell proliferation have been evaluated as potential predictive factors for chemotherapy (Corle *et al*, 1984; Willsher *et al*, 1998; Zambetti *et al*, 1999), but these tumour properties have so far shown little clinical utility. Recently many proteins including c-*erb*B-2, p53 and the *bcl-2* family have been investigated as predictors for chemosensitivity (Jacquemier *et al*, 1994; Muss *et al*, 1994; Sjöström *et al*, 2000; Sjöström, 2002a,b). However, none of these markers have shown clinical utility in predicting response for chemotherapy in advanced breast cancer.

Immunohistochemically measured microvessel density (MVD) has been applied to quantitate the angiogenesis in breast cancer. Results from most studies suggest that tumour microvessel density is associated with lymph node metastasis and worse outcome (Weidner *et al*, 1991; Bosari *et al*, 1992; Gasparini *et al*, 1994; Toi *et al*, 1995; Jacquemier *et al*, 1998; Hansen *et al*, 2000) although in some studies no association was seen (Van Hoef *et al*, 1993; Axelsson *et al*, 1995; Tynninen *et al*, 1999; Vincent-Salomon *et al*, 2001). It has also been suggested that MVD may be associated to endocrine treatment response in breast cancer (Gasparini *et al*, 1996).

In this study we investigated whether intratumoural microvessel density is associated with response to chemotherapy in patients with metastatic breast cancer. Earlier reports on MVD as a predictive marker for chemotherapy response in advanced breast cancer have not been published.

## MATERIALS AND METHODS

The patient population of the present study was a subgroup of 283 patients participating in a multicentre randomised trial comparing docetaxel with sequential methotrexate and 5-fluorouracil (MF) in advanced breast cancer (Sjöström *et al*, 1999). To enter the randomised study, patients were required to have histologically proven primary breast cancer that had progressed during or after first line anthracycline therapy for advanced disease or relapsed during or within 12 months after discontinuation of adjuvant anthracycline therapy. Ethical committees of all participating centres approved the study. Paraffin blocks were available for 134 patients. There was enough tumour tissue left in the blocks of 109 patients. Five patients were excluded because of excessive background staining with factor VIII antibody leaving 104 patients for final analysis (Table 1

**Table 1:**
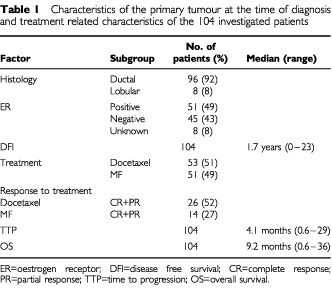
Characteristics of the primary tumour at the time of diagnosis and treatment related characteristics of the 104 investigated patients

).

Formalin fixed tissue samples were embedded in paraffin. For immunohistochemistry 5 μm thick sections were cut on coated slides. The slides were pretreated with 0.5% Trypsin and endogenous peroxidase was blocked. After rinsing the slides were incubated overnight at room temperature with diluted (1 : 10 000) polyclonal antibody against factor VIII related antigen (von Willebrand Factor, DAKO, Glostrup, Denmark) to stain capillary endothelium. Immunoperoxidase stainings were performed using a commercial avidin-biotin detection kit (Vectastain® Elite ABC Kit, Vector Laboratories, Burlingame, CA, USA) following the manufacturer's instructions. Bound peroxidase was visualised with 3-amino-9-ethylcarbazole. Finally the sections were counterstained in Mayer's haematoxylin.

Tumour microvessel density was determined from areas of highest vascularisation within invasive carcinoma. Microscopic slides were screened at low magnification (×20, ×40) to identify the most vascularised areas. Vessels were counted from the three most vascularised areas by one investigator (O Tynninen) at a magnification of ×200 (microscopic field area 0.785 mm^2^). An average of three counts reported in vessels per mm^2^ was used as the microvessel density of the tumour. The criteria introduced by Weidner *et al* (1991) were used to identify microvessels.

Response evaluation was performed according to the WHO recommendations (Miller *et al*, 1981). For statistical analysis clinical response was divided into two categories: response (complete and partial response) and non-response (stable disease and progression). Differences in treatment response were evaluated with the χ^2^ test in the whole patient population, and in the docetaxel and MF treated groups separately.

Disease free interval (DFI) was measured from the date of the primary diagnosis until the first recurrence of cancer, time to progression (TTP) from the date of randomisation till disease progression and overall survival (OS) from the date of randomisation till death. Kaplan–Meier plots were calculated for overall survival, disease free survival and time to progression. Differences in survival were tested with Cox logistic regression analysis with microvessel density as a continuous variable. Spearman correlation coefficients were calculated for MVD and several other previously assessed tumour related biological factors based on the same patient material and described elsewhere (Sjöström *et al*, 2000; Sjöström, 2002a,b). These factors included histological grade, MIB-1, expression of oestrogen receptor (ER), tumour suppressor protein p53, p21, mdm-2, the apoptosis related proteins *bcl-2*, bax, bcl-x_L_, bag-1, *fas*, *fas*L and the oncoprotein c-*erb*B-2. *P* values less than 0.01 for the correlation coefficients were considered significant. In the correlative analyses the significance level was set lower than the usual 0.05 because of the multiple comparisons.

## RESULTS

The median microvessel density of the tumours was 95.1 vessels per mm^2^ (range 32.3–247.9, 25th and 75th percentile 74.7 and 132.4, respectively. MVD was not significantly associated with response to chemotherapy when tested in the whole patient population (*P*=0.88) nor in separate groups of docetaxel (*P*=0.13) or MF (*P*=0.24) treated patients. However, low microvessel density showed a trend towards better response rate in the docetaxel arm (RR 68% *vs* 40%, Table 2

**Table 2:**
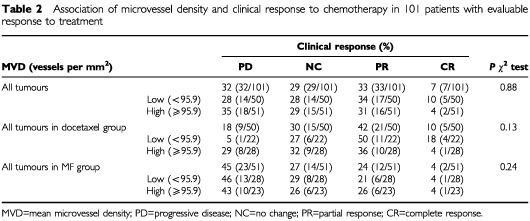
Association of microvessel density and clinical response to chemotherapy in 101 patients with evaluable response to treatment

). Overall survival or time to tumour progression were not associated with MVD in univariate analysis neither in the whole patient population (*P*=0.75) nor in docetaxel (*P*=0.64) or MF (*P*=0.88) groups. However, disease free interval from primary diagnosis to the appearance of first metastases was shorter in patients with tumour MVD above median (*P*=0.01, Figure 1

**Figure 1:**
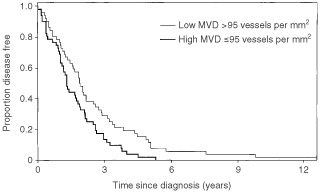
Kaplan–Meier plot showing disease free interval in 104 breast cancer patients. Disease free interval was shorter in patients with MVD above median 95 vessel per mm^2^ (*P*=0.01).

).

We also assessed the correlation between MVD and other tumour biological factors previously studied in the same population (Table 3

**Table 3:**
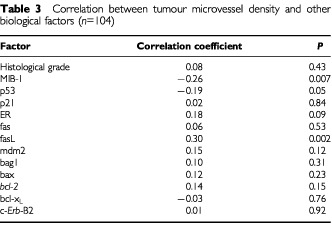
Correlation between tumour microvessel density and other biological factors (*n*=104)

). MVD was inversely correlated with MIB-1 proliferation index (*P*=0.007). Tumour grade did not correlate with MVD. Expression of fas ligand (fasL) was positively correlated with MVD (*P*=0.002) but no correlation was found with any other biological factor investigated (Table 3).

## DISCUSSION

We studied intratumoural microvessel density in 104 patients with advanced breast cancer to evaluate its predictive value for chemotherapy. We could not demonstrate any difference in response to chemotherapy, overall survival or time to progression between the patient groups according to vascular density either in the whole patient material or in the docetaxel arm. However, in the MF arm there was a trend towards better chemotherapy response in the low microvessel density group but the difference was not statistically significant. High MVD was associated with short disease free interval which is in line with several previous studies (Weidner *et al*, 1992; Toi *et al*, 1993; Hansen *et al*, 2000).

Preclinical studies suggest that chemoresistance might be associated with tumour vascularity (Gasparini *et al*, 1996). Instead of facilitating the access of chemotherapeutic agents into the tumour neovascularisation has been proposed to inhibit penetration of cytotoxic drugs into tumour tissue due to increased interstitial pressure (Folkman, 1995). Consequently, one would expect that response to chemotherapy in more vascularised tumours would be weaker compared to those with low MVD which is in accordance with our findings in the docetaxel group.

Against our expectations response to docetaxel was not related to microvessel density. Previous reports suggest that taxanes including docetaxel used in this study also harbour an anti-angiogenic effect themselves (Belotti *et al*, 1996; Sweeney *et al*, 2001). However, in the present study, the methodology to test the predictive value of MVD for response to taxanes was not optimal. MVD and other parameters were counted in the primary tumour and the measured outcome was clinical response after disease recurrence which may be a source of error because the biological properties of metastases may differ from the primary tumour.

To the best of our knowledge there are no previous report on the predictive utility of MVD for chemotherapy in advanced breast cancer. Two studies have investigated microvessel density in breast cancer patients receiving either adjuvant or neoadjuvant chemotherapy. In one study Protopapa *et al* (1993) demonstrated that the patients who survived longer after mastectomy and chemotherapy had higher MVD. In the other study Paulsen *et al* (1997) did not find any difference in the outcome according to MVD in patients treated with neoadjuvant chemotherapy.

We also tested correlations between microvessel density and several other tumour biological factors (histological grade, MIB-1, ER, p53, p21, mdm-2, *bcl-2*, bax, bcl-x_L_, bag-1, *fas*, *fas*L and c-*erb*B-2). Only two significant correlations were seen. We noted an inverse correlation between MVD and MIB-1 proliferation which is in line with the recent findings by another group (Medri *et al*, 2000). However, most studies have not found significant association between MVD and MIB-1 proliferation index (Weidner *et al*, 1992; Vartanian and Weidner, 1994; Jacquemier *et al*, 1998; Tynninen *et al*, 1999).

The positive correlation between vascular density and *fas* ligand expression was particularly interesting. The fas receptor belongs to the family of tumour necrosis factor related death receptors and fasL is its corresponding ligand. Binding of a death ligand to its receptor activates one major apoptotic pathway. Results from some preclinical studies suggest that classic anticancer drugs may need fas and fasL to induce apoptosis. Interestingly, stimulation of fas receptor by agonistic anti-fas monoclonal antibodies also promotes angiogenesis in murine model (Biancone *et al*, 1997). Thus, fas receptor stimulation by fasL might induce angiogenesis in breast cancer as well. These findings may explain why fasL expression was correlated with angiogenesis in our material. Further studies are required to confirm the possible role of apoptotic fas/fasL system in tumour neovascularisation.

In conclusion the results of this study suggest that microvessel density of primary tumour is not useful in predicting response to taxane or 5-fluorouracil based chemotherapy in advanced breast cancer.
